# Household income and county income inequality are associated with financial hardship among cancer survivors in New Jersey

**DOI:** 10.1007/s11764-024-01730-z

**Published:** 2024-12-11

**Authors:** Irina B. Grafova, Katie A. Devine, Shawna V. Hudson, Denalee O’Malley, Lisa E. Paddock, Elisa V. Bandera, Adana A. M. Llanos, Angela J. Fong, Andrew M. Evens, Sharon Manne

**Affiliations:** 1 Edward J. Bloustein School of Planning and Public Policy, Rutgers University, New Brunswick, NJ USA; 2 Rutgers Cancer Institute of New Jersey, New Brunswick, NJ USA; 3 Department of Family Medicine and Community Health, Johnson Medical School, Rutgers Robert Wood, New Brunswick, NJ USA; 4 Department of Epidemiology, Mailman School of Public Health and Herbert Irving Comprehensive Cancer Center, Columbia University Irving Medical Center, New York, NY USA; 5 School of Kinesiology and Rogel Cancer Center, University of Michigan, Ann Arbor, MI USA

**Keywords:** Financial hardship, Financial burden, Income, Income inequality, Foregoing care

## Abstract

**Purpose:**

To examine how household income and county income inequality are linked to financial hardship among cancer survivors.

**Methods:**

Cancer survivors (*n* = 864) identified through the New Jersey State Cancer Registry were surveyed from August 2018 to January 2022. Local area income inequality was reflected by the Gini index a measure of income inequality at the county level. Multivariable logistic regression analyses were performed, and the average marginal effect (AME) was calculated.

**Results:**

Compared to survivors residing in households with income of $90,000 or more (higher income), those with household incomes between $50,000 and $89,999 (middle income) had a significantly higher risk of ever being unable to cover their share of the cost of cancer-related medical care (AME = .104, *p* = .001), higher risk of foregoing care in the past 12 months because of cost, including dental care (AME = .124, *p* < .001), eye care (AME = .082, *p* = .005), and mental health care or counseling (AME = .067, *p* = .002). An increase in the Gini index from the 25th to 75th percentile was associated with an increased risk of unmet needs in paying for follow-up care or medications related to cancer (AME = .021, *p* = .014) and an increased risk of foregoing doctor visits (AME = .017, *p* = .02) and eye care (AME = .03, *p* = .002) because of cost in the past 12 months.

**Conclusions:**

Local area income inequality was associated with certain aspects of cancer survivors’ experience of financial hardship.

**Implications for cancer survivors:**

It is important to consider refining and extending financial navigation programs to survivors residing in areas with high income inequality.

## Introduction

Cancer survivors experience financial hardships as a direct result of their treatment [1]. Between 12 and 43% of cancer survivors are unable to cover their share of cancer-related medical costs, and between 21 and 85% worry about paying large medical bills [1–4]. In an effort to pay for their cancer-related medical costs, cancer survivors may forego or delay cancer-related follow-up care [5] and/or medical care unrelated to their cancer [6–8], including general doctor visits [6, 9], filling prescriptions [7, 8, 10–12], dental care [8, 11], mental health care [7, 8, 11], and eye care [11].

Although there is extensive literature [13] documenting a strong association between lower income and greater worry about paying for cancer care [1, 14, 15], greater difficulty paying for cancer care [10, 14], and/or increased likelihood of delaying or foregoing non-cancer-related medical care [1, 2, 5, 10, 15, 16], less attention has been paid to financial hardship among middle-income cancer survivors [14, 15]. Evaluating the financial hardships experienced by these survivors is important because evidence from the general US population has found that middle-income households are at higher risk of medical financial hardship [17]. Previous cancer survivor studies have grouped middle- and high-income individuals in analyses [2], treated income as a continuous variable [10], focused on a single cancer diagnosis [16], or did not include income in the analysis [11]. The one study that included middle income as a separate variable did not report a significant relationship between middle income and financial hardship [3].

Understanding the role of income inequality as a contributor to financial hardship among cancer survivors may be important. Income inequality reflects the extent to which income is distributed unevenly in a community and has been referred to as the gap between the rich and poor. Research conducted outside of cancer care has found that higher income inequality is associated with financial hardship [18] because inequality leads to greater social differentiation. Social differentiation may reduce social cohesion and trust and increase stress and anxiety in comparison to those with more financial resources [19, 20]. Thus, living in an area of higher income inequality not only can exacerbate psychological aspects of financial hardship but also can have a detrimental effect on coping with financial hardship by reducing reliance on community resources [18, 19]. In this way, income inequality at the area level represents a social/structural driver of health. Unfortunately, income inequality has increased dramatically over the past four decades, while inflation-adjusted incomes of low- and middle-income households have barely changed since the 1970s [21]. To our knowledge, income inequality has not been included in the characterization of financial hardship among cancer survivors.

To better understand the role of income and income inequality in financial hardship experienced by cancer survivors, we conducted a statewide study of cancer survivors in New Jersey (NJ). Income inequality in New Jersey is very close to the national average, while median household income is above the national average [22]. While most studies use either national population-based surveys or data centered around particular providers or provider networks, there is a sparsity of statewide evidence on financial hardship among cancer survivors [13, 23]. Statewide studies are better equipped than studies centered around particular providers or provider networks to include cancer survivors from communities with a wide range of income inequality. Statewide studies are also better able to inform state-level policy and practice than national studies. Following Altice et al.’s framework [13], we examined several financial hardship measures that reflected material conditions, psychological response, and coping behavior domains of financial hardship. We had two main hypotheses. First, we hypothesized that middle-income survivors would be more likely to experience financial hardship than high-income survivors. Second, we hypothesized that greater income inequality in the county in which a survivor resides would be associated with greater financial hardship.

## Methods

### Eligibility

Eligibility criteria included individuals who were (a) 18–85 years of age; (b) a current resident of NJ; (c) diagnosed in 2015 or 2016 with a primary case of genitourinary (i.e., bladder and prostate), female breast, gynecologic (i.e., cervical, endometrial, ovarian), colorectal, lung, melanoma, or thyroid cancer; and (d) able to read and speak English. Cases were assigned a random number, and a sample was constructed that represented the incidence and population characteristics (race, ethnicity, sex) of each county.

### Procedures and participation

The study received institutional review board approval from the Rutgers Institutional Review Board prior to study commencement and conforms to recognized standards of US Federal Policy for the Protection of Human Subjects. Potentially eligible participants were identified through the New Jersey State Cancer Registry (NJSCR), and eligibility was confirmed by an Oncology Data Specialist (ODS). NJSCR staff mailed a recruitment package to prospective participants, which included a cover letter, study information, questionnaire, and postage-paid return envelope. One week after the recruitment package was mailed, individuals were called by NJSCR staff to confirm receipt and to answer any questions about the study. Interested patients completed the questionnaire and mailed it back to the host institution. Prospective participants were called by telephone 1–2 times per week on varying days and times to increase the likelihood of patient contact and participation, with a maximum of 8 calls; those who could not be contacted were considered passive refusers. When reached, individuals who did not agree to participate were considered active refusers. Additionally, recruitment packets were resent to non-responders up to three times during the recruitment period. A returned, completed questionnaire was considered to be an individual’s consent for study inclusion per Rutgers Institutional Review Board written informed consent waiver approval. Participants who returned a completed survey received a $25 gift card in appreciation of their time spent.

Between August 2018 and January 2022, there were 3346 individuals who met the initial eligibility requirements and were contacted for study participation. Among them, 538 were deemed ineligible, 1830 refused, 116 could not be contacted (due to incorrect address or telephone number), and 864 returned the survey (31.9% response rate). Comparisons of the 864 acceptors and the 1830 refusers based on available data (sex, race, ethnicity, age, cancer type, cancer stage) indicated only two differences: Hispanic survivors were more likely to decline participation (75.5%) than non-Hispanic survivors (67.5%) (Chi-square = 4.4, *p* < 0.05) and there were significant differences based on type of cancer (Chi-square = 20.4, *p* < 0.001), with breast cancer survivors having the lowest refusal rate (61.8%) and thyroid cancer survivors having the highest refusal rate (74.8%).

### Financial hardship measures

We classified financial hardship measured into three domains: material conditions, psychological response, and coping behaviors [13]. Material conditions measures included difficulty paying for cancer care and unmet need in paying for non-medical and medical expenses. To assess difficulty paying for cancer care, items asked participants to report if they were ever unable to cover their share of the cost of medical care visits for cancer, its treatments, or the lasting effects of that treatment (*yes*, *no*). To assess unmet needs in paying for non-medical and medical expenses, items asked participants to report their level of financial support needed for help with (a) paying non-medical bills related to cancer, such as travel, accommodations, and special foods and (b) paying for follow-up care and/or medications related to cancer. The responses included three options: *a need has been met*, *a need has not been met*, and *hasn’t been a need at all*. For the analyses, responses of met need or no need were used as the reference category.

The psychological response measure reflected if participants reported that they ever worried about having to pay large medical bills related to cancer (*yes*, *no*).

Coping behavior measures reflected foregoing or delaying cancer or non-cancer medical care because of cost. Five questions asked respondents if there was a time in the past 12 months when they needed (1) a doctor visit, (2) medications, (3) dental care, (4) eye care (or eyewear), or (5) mental health care or counseling but did not get this care because they could not afford it. Based on responses to these items, five dichotomous measures of foregoing care because of cost were created. For the analyses, not forgoing care because of cost served as the reference category. Responses of “Don’t know” and “Prefer not to answer” were treated as missing data and dropped from the analytic sample.

### Household income and local area income inequality measures

#### Household income

Participants reported their household income (less than $10,000; $10,000 to $19,999; $20,000 to $29,999; $30,000 to $39,999; $40,000 to $49,999; $50,000 to $59,999; $60,000 to $69,999; $70,000 to $79,999; $80,000 to $89,999; $90,000 or more). Households with income less than $50,000; $50,000–$89,999; and $90,000 or more were classified as lower income, middle income, and higher income, respectively. The middle-income category $50,000$89,000 roughly corresponds to the second quartile of the household income distribution in New Jersey at the time of the survey. Local area income poverty was reflected by the percentage of persons below poverty in the respondent’s census tract of residence based on 2014–2018 American Community Survey data [24].

#### Income inequality

Local area income inequality was reflected by the Gini index of income inequality in a county of residence based on 2017–2020 American Community Survey data [22]. The Gini index measures the extent to which the distribution of income within counties deviates from a perfectly equal distribution. A Gini index of zero represents perfect income equality, and an index of 100 implies the highest level of income inequality.

### Other predictors included in the analyses

#### Sociodemographic and cancer-related variables

Participants self-reported age, sex (male, female), race and ethnicity (white non-Hispanic, Black non-Hispanic, other race non-Hispanic, Hispanic), marital status (married, not married), number of comorbidities, and educational attainment (college degree and no college degree). Cancer diagnosis and cancer treatment: Participants were asked to report the type and date of their first primary cancer diagnosis and report treatments they received (surgery, chemotherapy, radiation).

#### Mobility limitation

Participants who reported difficulty with walking several blocks because of a health problem or difficulty with pulling or pushing large objects like a living room chair because of a health problem were categorized as having mobility limitation [25].

#### Health insurance

Survey data was merged with the NJSCR data on health insurance coverage at the time of diagnosis and was categorized as private, Medicare, Medicaid, or other public health insurance coverage.

#### Number of physical health comorbidities

This includes diabetes; asthma; high blood pressure; emphysema or chronic bronchitis; heart disease, angina, or heart attack; high cholesterol; kidney problems; peripheral vascular disease; cerebrovascular disease; dementia; connective tissue disease; liver disease; and AIDS.

#### Number of mental health comorbidities

This includes depression, anxiety disorder, schizophrenia, bipolar disorder, and post-traumatic stress disorder.

### Statistical approach

Sample characteristics are presented as frequencies and proportions. The percentage of missing data was calculated for each variable. We used multinomial logistic regression to explore how difficulty paying for cancer care and foregoing care outcomes were associated with demographic, socio-economic, and other factors listed above, including the following covariates: age, sex, ethnicity, race, marital status, education, type of cancer treatment, health insurance coverage, household income, local area poverty, local area Gini income inequality index, number of physical health comorbidities, number of mental health comorbidities, the mobility limitations indicator, and year of survey dummy variables (i.e., 1 for particular year, 0 for not). Collinearity diagnostic was performed to assess for potential multicollinearity in variables, and variance inflation factor (VIF) was calculated with VIF < 5 indicating no obvious collinearity [26].

For each outcome, we excluded observations with missing data on the outcome variable or missing values for marital status, year of the survey, education, and ethnicity. Missing data on age, sex, and race was imputed based on the data from NJSCR, with 1.4% for age, 0.1% for sex, and 3.1% for race. Given that data on household income, type of treatment, health insurance, number of comorbidities, and presence of mobility limitations were missing in > 5% of cases, dummy variables indicating that the response was missing were included in the analysis. To assist in the interpretation of the multinomial logistic regression estimation results, we conducted a post-estimation transformation and calculated the average marginal effect (AME) of each independent variable on the probability of the outcome using the margins procedure in Stata. The AME reflects the difference in average model-adjusted predicted probability conditional on all observations being in a category and average predicted probability conditional on all observations being in the reference category. Statistical analyses were performed with StataMP 17 (StataCorp LP; College Station, TX, USA). We compared the participants and non-participants with regard to age at diagnosis, sex, and Hispanic ethnicity and found no statistically significant differences (at *p*-value = 0.05) between the two groups. We found that participants were more likely to be non-Hispanic White (75.5% versus 66.8%) and less likely to be non-Hispanic Black (12.5% versus 17.5%) compared to non-responders.

## Results

### Descriptive information

Sample characteristics are shown in Table 1. More than half of the sample was comprised of females (59.1%) and married individuals (65.4%). Seventy-five percent of the sample self-identified as non-Hispanic White (75.2%). About half of the participants earned a college degree. In terms of medical insurance, 47.2% were covered by private health insurance, 36.7% were covered by Medicare, and 4.1% were covered by Medicaid. The most common cancers reported were breast (24.3%), prostate (17.5%), and colon (11.6%). Most participants (74.0%) received surgical treatment; of those who received surgery, 48.3% also received chemotherapy and/or radiation. The average participant had been diagnosed with cancer 3 years ago.

**Table 1:** Sample characteristics (*N* = 864)

	%^f^	*N*
Sex		
Female	59.1	511
Male	40.9	353
Age, mean (range 20–87)	63.6	864
Race and ethnicity^a^		
White non-Hispanic	75.2	649
Black non-Hispanic	12.6	109
Other race non-Hispanic	7.3	63
Hispanic	6.3	54
Missing	0.1	1
Marital status		
Married	65.4	565
Not married	33.0	285
Missing	1.6	14
Education		
College degree	51.2	448
No college degree	46.3	400
Missing	1.9	16
Gini, mean	46.4	862
Census tract poverty rate, mean	8.2	862
Annual household income		
< $49,999	23.7	205
$50,000–$89,999	22.1	191
$90,000 or more	30.8	266
Missing	23.4	202
Treatment		
Surgery	38.2	330
Surgery, radiation, and chemotherapy	12.9	111
Surgery and chemotherapy	10.4	90
Surgery and radiation	12.5	108
Other treatment^b^	12.2	123
Missing	11.8	102
Type of cancer		
Breast	24.3	210
Colon	11.6	100
Lung	9.6	83
Prostate	17.5	151
Thyroid	7.2	62
Urinary bladder	9.0	78
Melanoma	8.8	76
Other cancer	12.0	104
Years since diagnosis, mean	3.0	864
Health insurance		
Private	47.2	408
Medicare	36.7	317
Medicaid	4.1	35
Other public insurance	6.6	57
Missing^c^	5.4	47
Number of physical health conditions^d^, mean	1.6	797
Missing number of physical health conditions	7.75	67
Number of mental health conditions^e^, mean	0.3	831
Number of mental health conditions, missing	3.8	33
Year of the survey		
2018	17.6	152
2019	22.0	190
2020	15.2	131
2021	45.3	391

^a^10 respondents indicated two races and 1 respondent indicated three races

^b^ “Other treatment” includes 10 respondents who received chemotherapy and no surgery or radiation therapy; 26 respondents who received chemo and radiation therapies; 32 respondents who reported receiving no chemo, no surgery, and no radiation treatment; 54 respondents who received radiation therapy but no surgery or chemotherapy

^c^Includes 12 respondents who were uninsured at the time of diagnosis

^d^Physical health conditions include diabetes; asthma; high blood pressure; emphysema or chronic bronchitis; heart disease, angina, or heart attack; high cholesterol; kidney problems; peripheral vascular disease; cerebrovascular disease; dementia; connective tissue disease; liver disease; and AIDS

^e^Mental health conditions include depression, anxiety disorder, schizophrenia, bipolar disorder, and post-traumatic stress disorder

^f^For continuous variables, means, not proportions, are presented

With regard to household income, approximately 24% of survivors reported an annual household income of less than $50,000, and 22.1% reported a household income of $50,000–$89,999. The mean census tract poverty rate was 8.2%. The average Gini income inequality index was 46.4. The Gini income inequality interquartile range was 44.7–47.6, indicating a redistribution of 2.9% of total county income away from poorer to richer households between the 25th and 75th percentiles. Descriptive information about financial hardships is shown in Table 2. A total of 12.3% of the sample reported that they were unable to cover their out-of-pocket share of cancer care costs. About one out of ten survivors reported an unmet need in paying non-medical bills related to cancer (10.5%), and about 9% reported an unmet need in paying for follow-up care and/or medications related to cancer (9.1%). Nearly a third of the sample (32.9%) reported that they worried about having to pay large medical bills related to cancer. The most frequent types of health care services cancer survivors reported foregoing due to finances were dental care (15.4%) and eye care (11.3%). About 7.3% of the sample reported that they did not obtain medications when needed because of cost, 5.9% reported foregoing visits to a doctor, and 4.4% reported foregoing mental health care.

**Table 2:** Financial hardship outcomes (*N* = 864)

Variable	%	*N*
Material conditions		
Ever been unable to cover out-of-pocket share of the cancer care cost		
Yes	12.3	106
No	86.0	743
Missing	1.7	15
Current^a^ unmet need in paying non-medical bills related to cancer		
Yes	10.5	91
No	87.5	756
Missing	2.0	17
Current^a^ unmet need in paying for follow-up care and/or medications related to cancer		
Yes	9.1	79
No	89.0	769
Missing	1.9	16
Psychological response		
Ever worried about having to pay large medical bills related to cancer		
Yes	32.9	284
No	65.9	569
Missing	1.2	11
Coping behaviors		
Foregoing care in the past 12 months because of cost		
Dental care		
Yes	15.4	133
No	81.5	704
Missing	3.1	27
Eye care (including eyeglasses)		
Yes	11.3	98
No	85.1	735
Missing	3.6	31
Medications		
Yes	7.3	63
No	89.5	773
Missing	3.2	28
Doctor visit		
Yes	5.9	51
No	91.4	790
Missing	2.7	23
Mental health care		
Yes	4.4	38
No	88.8	767
Missing	6.8	59

^a^Unmet need in the last month

### Predictors of material condition and psychological response measures of financial hardship

Table 3 shows that, compared to survivors residing in households with income of $90,000 or more (higher income), those with household incomes between $50,000 and $89,999 (middle income) had a significantly higher risk of ever being unable to cover their share of the cost of cancer-related medical care (AME = 0.104, *p* = 0.001) and higher risk of ever worried about having to pay large medical bills related to cancer (AME = 0.122, *p* = 0.004). Similarly, compared to survivors residing in higher-income households, those with household incomes under $50,000 had a significantly higher risk of ever being unable to cover their share of the cost of cancer-related medical care (AME = 0.123, *p* = 0.001) or having unmet need in paying for follow-up care or medications related to cancer (AME = 0.077, *p* = 0.010) and higher risk of ever worried about having to pay large medical bills related to cancer (AME = 0.167, *p* < 0.001). Living in a higher income inequality area was significantly associated with an increased risk of difficulty paying for cancer care. Table 3 shows that an increase in the Gini index of income inequality from the 25th to 75th percentile is associated with an increased risk of unmet needs in paying for follow-up care or medications related to cancer (AME = 0.021, *p* = 0.014) and increased risk of having ever worried about having to pay large medical bills related to cancer (AME = 0.037, *p* = 0.011). Recall, that an increase in the Gini index of income inequality from the 25th to 75th percentile in the context of New Jersey indicates redistribution of 2.9% of total county income away from poorer to richer households. The relationship between census tract poverty rate and difficulty paying for cancer care outcomes was not statistically significant.

**Table 3:** Material conditions and psychological response measures of financial hardship logistic regressions results

	Ever unable to cover cancer care OOP (*N* = 824)		Unmet need in paying non-medical bills related to cancer (*N* = 821)		Unmet need in paying for follow-up/meds related to cancer (*N* = 822)		Ever worried about having to pay large cancer medical bills (*N* = 828)	
	AME (95% CI)	*p*	AME (95% CI)	*p*	AME (95% CI)	*p*	AME (95% CI)	*p*
Gini^a^	.014 (− .006; .035)	.172	.006 (− .013; .025)	.524	.021 (.004; .038)	.014	.037 (.009; .065)	.011
Census tract poverty rate^b^	− .002 (− .02; .016)	.820	.007 (− .008; .021)	.366	.01 (− .004; .024)	.148	.005 (− .023; .033)	.720
Annual household income								
< $49,999	.123 (.054; .192)	.001	.057 (− .005; .119)	.070	.077 (.018; .135)	.010	.167 (.068; .266)	.001
$50,000–$89,999	.104 (.043; .164)	.001	.044 (− .013; .102)	.131	.049 (− .003; .100)	.066	.122 (.039; .204)	.004
≥ $90,000, reference								
Missing	.027 (− .025; .08)	.311	.018 (− .038; .073)	.531	.036 (− .015; .087)	.171	.083 (− .001; .166)	.052
Mobility limitation								
No reference								
Yes	.109 (.055; .164)	< .001	.103 (.052; .153)	< .001	.067 (.017; .117)	.009	.192 (.107; .277)	< .001
Missing	.029 (− .032; .09)	.345	.109 (.061; .158)	< .001	.087 (.039; .135)	< .001	.106 (.022; .191)	.014
Female	− .016 (− .067; .035)	.53	− .038 (− .082; .007)	.100	− .042 (− .087; .002)	.062	− .026 (− .097; .044)	.459
Age	− .004 (− .007; − .002)	.001	− .003 (− .005; − .001)	.003	− .004 (− .006; − .002)	.001	− .008 (− .012; − .005)	< .001
Race and ethnicity								
White non-Hispanic reference								
Hispanic	.056 (− .029; .142)	.194	.095 (.026; .164)	.007	.03 (− .042; .102)	.412	− .037 (− .175; .101)	.602
Black non-Hispanic	.049 (− .013; .111)	.121	.044 (− .011; .099)	.114	− .004 (− .06; .052)	.888	.010 (− .092; .112)	.842
Other race non-Hispanic	.084 (.012; .156)	.022	.104 (.038; .171)	.002	.075 (.01; .14)	.024	.130 (.019; .240)	.021
Married	.007 (− .04; .055)	.765	.011 (− .033; .055)	.616	− .013 (− .055; .029)	.541	.112 (.041; .182)	.002
College degree	− .041 (− .087; .006)	.086	− .081 (− .125; − .036)	< .001	− .062 (− .105; − .019)	.005	− .014 (− .079; .050)	.66
Treatment type								
Surgery reference								
Surgery, radiation, and chemotherapy	.067 (0; .134)	.05	.063 (.004; .121)	.036	.097 (.038; .156)	.001	.118 (.025; .212)	.013
Surgery and chemotherapy	.083 (.015; .151)	.017	.08 (.022; .139)	.007	.104 (.044; .164)	.001	.154 (.056; .252)	.002
Surgery and radiation	.047 (− .024; .119)	.194	.023 (− .045; .091)	.511	.065 (− .003; .133)	.06	.077 (− .018; .173)	.113
Other treatment	.076 (.004; .149)	.038	.051 (− .015; .116)	.132	.081 (.015; .147)	.016	.062 (− .037; .161)	.217
Missing	.063 (− .009; .136)	.085	.037 (− .028; .102)	.268	.052 (− .016; .120)	.136	.020 (− .084; .124)	.711
Health insurance								
Private reference								
Medicare	− .034 (− .093; .026)	.27	− .065 (− .119; − .011)	.019	− .034 (− .086; .017)	.192	− .099 (− .182; − .016)	.020
Medicaid	− .106 (− .214; .002)	.055	− .004 (− .078; .069)	.905	− .046 (− .125; .033)	.252	− .387 (− .580; − .194)	< .001
Other public insurance	0 (− .084; .083)	.991	.059 (− .007; .125)	.082	− .015 (− .09; .060)	.693	.086 (− .031; .204)	.149
Missing	− .001 (− .095; .094)	.987	− .109 (− .231; .013)	.079	− .046 (− .144; .052)	.36	− .201 (− .358; − .045)	.012
Number of physical health conditions	.006 (− .012; .024)	.508	.009 (− .007; .024)	.286	.005 (− .011; .021)	.534	.002 (− .024; .027)	.884
Missing number of physical health conditions	.044 (− .043; .13)	.321	.029 (− .05; .109)	.466	.016 (− .063; .096)	.685	.080 (− .052; .211)	.235
Number of mental health conditions	.01 (− .017; .037)	.477	− .013 (− .038; .012)	.317	− .017 (− .043; .009)	.195	− .006 (− .048; .036)	.773
Missing number of mental health conditions	.013 (− .106; .131)	.834	− .111 (− .275; .054)	.186	− .096 (− .251; .06)	.227	.113 (− .053; .280)	.182
Year of the survey								
2018 reference								
2019	− .021 (− .086; .045)	.536	− .048 (− .107; .011)	.108	− .010 (− .068; .048)	.733	− .006 (− .106; .094)	.908
2020	− .052 (− .132; .027)	.199	− .035 (− .102; .032)	.308	− .019 (− .085; .047)	.568	− .032 (− .143; .080)	.580
2021	− .051 (− .109; .007)	.087	− .059 (− .111; − .007)	.026	− .050 (− .103; .002)	.060	.030 (− .058; .117)	.507

Note: all models are estimated as multivariate logistic regressions

^a^AME of going from 25 to 75th percentile of Gini

^b^AME of going from 25 to 75th percentile of census tract poverty rate

### Predictors of coping behavior measures of financial hardship

Table 4 shows that compared to survivors residing in higher-income households, those residing in middle-income households had a higher risk of foregoing care in the past 12 months because of cost, including dental care (AME = 0.124, *p* < 0.001), eye care (or eyewear) (AME = 0.082, *p* = 0.005), and mental health care or counseling (AME = 0.067, *p* = 0.002). Similarly, compared to survivors residing in higher-income households, those with household incomes under $50,000 had higher risk of foregoing care in the past 12 months because of cost, including doctor visits (AME = 0.071, *p* = 0.016), prescription medications (AME = 0.077, *p* = 0.007), dental care (AME = 0.145, *p* < 0.001), eye care (or eyewear) (AME = 0.136, *p* < 0.001), and mental health care or counseling (AME = 0.043, *p* = 0.039). An area of higher income inequality was associated with an increased risk of foregoing care because of cost. Table 4 shows that an increase in the Gini index of income inequality from the 25th to 75th percentile is associated with an increased risk of foregoing doctor visits (AME = 0.017, *p* = 0.02) and eye care (AME = 0.03, *p* = 0.002) because of cost in the past 12 months. The relationship between census tract poverty rate and foregoing care because of cost in the past 12 months was not statistically significant.

**Table 4:** Coping behavior measures of financial hardship logistic regression results: foregoing care due to cost in the past 12 months

Predictor	Doctor		Medications		Dental care		Eye care or glasses/contacts		Mental health care or counseling	
	*N* = 816		*N* = 812		*N* = 812		*N* = 809		*N* = 783	
	**AME (95% CI)**	***p***	**AME (95% CI)**	***p***	**AME (95% CI)**	***p***	**AME (95% CI)**	***p***	**AME (95% CI)**	***p***
Gini^a^	.017 (.003; .031)	.02	.012 (− .005; .029)	.155	.013 (− .01; .036)	.269	.03 (.011; .05)	.002	.006 (− .007; .019)	.386
Census tract poverty rate^b^	0 (− .012; .012)	.989	.007 (− .005; .019)	.268	− .005 (− .026; .015)	.597	− .004 (− .022; .015)	.705	.003 (− .01; .016)	.614
Annual income										
< $49,999	.071 (.013; .128)	.016	.077 (.021; .134)	.007	.145 (.07; .22)	< .001	.136 (.065; .207)	< .001	.043 (.002; .084)	.039
$50,000–$89,999	.002 (− .037; .04)	.923	.034 (− .011; .079)	.140	.124 (.059; .188)	< .001	.082 (.025; .138)	.005	.067 (.025; .11)	.002
≥ $90,000, reference										
Missing	− .007 (− .044; .030)	.694	.013 (− .03; .056)	.552	.06 (0; .121)	.051	.024 (− .026; .074)	.348	.017 (− .014; .048)	.290
Mobility limitation										
No reference										
Yes	.048 (.007; .090)	.023	.066 (.022; .11)	.003	.125 (.066; .184)	< .001	.073 (.016; .13)	.012	.016 (− .027; .058)	.471
Missing	.048 (.007; .089)	.022	.055 (.008; .101)	.021	.055 (− .007; .117)	.081	.08 (.025; .134)	.004	.027 (− .008; .061)	.132
Female	− .026 (− .063; .011)	.164	− .037 (− .076; .003)	.069	.016 (− .04; .071)	.582	− .004 (− .055; .048)	.887	.048 (.001; .094)	.043
Age	− .003 (− .005; − .001)	.001	− .002 (− .004; 0)	.03	− .003 (− .006; 0)	.051	− .003 (− .006; − .001)	.015	− .001 (− .003; 0)	.077
Race and ethnicity										
White non-Hispanic reference										
Hispanic	− .029 (− .107; .049)	.471	.001 (− .074; .076)	.97	− .035 (− .154; .085)	.570	− .064 (− .173; .045)	.251	− .051 (− .122; .02)	.162
Black non-Hispanic	− .013 (− .060; .034)	.582	− .013 (− .066; .039)	.615	.038 (− .032; .108)	.288	− .015 (− .081; .051)	.655	− .08 (− .162; .002)	.055
Other race non-Hispanic	.107 (.060; .155)	< .001	.067 (.004; .129)	.036	.088 (.008; .168)	.031	.08 (.010; .150)	.025	− .015 (− .072; .042)	.602
Married	− .027 (− .062; .007)	.122	.006 (− .034; .045)	.775	− .021 (− .072; .030)	.418	− .04 (− .086; .006)	.089	− .016 (− .045; .014)	.298
College degree	− .033 (− .069; .004)	.078	− .082 (− .126; − .037)	< .001	− .064 (− .114; − .014)	.012	− .031 (− .077; .015)	.180	.022 (− .008; .052)	.146
Treatment type										
Surgery reference										
Surgery, radiation, and chemotherapy	.020 (− .027; .068)	.405	.027 (− .029; .083)	.339	.056 (− .012; .124)	.108	.047 (− .017; .111)	.147	.032 (− .005; .07)	.089
Surgery and chemotherapy	.040 (− .005; .086)	.084	.046 (− .007; .098)	.087	− .011 (− .09; .067)	.778	.023 (− .047; .092)	.525	− .017 (− .07; .036)	.534
Surgery and radiation	− .001 (− .056; .055)	.979	.016 (− .044; .076)	.607	− .022 (− .098; .054)	.574	.063 (− .001; .128)	.055	− .027 (− .077; .023)	.298
Other treatment	.018 (− .038; .073)	.529	.006 (− .053; .065)	.853	− .049 (− .134; .035)	.254	.014 (− .061; .089)	.717	.02 (− .035; .075)	.474
Missing	.007 (− .045; .059)	.791	.021 (− .036; .078)	.476	− .033 (− .117; .05)	.433	− .019 (− .1; .062)	.639	.003 (− .049; .055)	.913
Health insurance										
Private reference										
Medicare	− .035 (− .08; .01)	.125	− .014 (− .06; .032)	.544	− .037 (− .099; .026)	.251	− .033 (− .09; .024)	.261	− .005 (− .046; .035)	.798
Medicaid	− .017 (− .078; .044)	.588	− .061 (− .139; .018)	.129	− .232 (− .38; − .084)	.002	− .078 (− .181; .025)	.137	.017 (− .05; .084)	.616
Other public insurance	− .034 (− .1; .033)	.322	− .018 (− .09; .055)	.63	− .098 (− .207; .011)	.079	− .036 (− .121; .05)	.412	− .021 (− .08; .038)	.492
Missing	.002 (− .065; .068)	.961	− .007 (− .088; .075)	.875	− .118 (− .256; .02)	.095	− .122 (− .259; .014)	.079	− .038 (− .12; .045)	.369
# physical health conditions	.008 (− .005; .021)	.223	.009 (− .004; .023)	.177	.009 (− .011; .028)	.374	.012 (− .006; .03)	.193	− .007 (− .02; .005)	.25
Missing # physical health conditions	.015 (− .047; .077)	.629	.039 (− .028; .106)	.258	.093 (.003; .183)	.043	.073 (− .012; .157)	.091	− .033 (− .115; .048)	.422
# mental health conditions	− .032 (− .06; − .004)	.027	− .007 (− .029; .015)	.549	.013 (− .017; .044)	.384	− .014 (− .043; .014)	.330	.018 (.002; .033)	.026
Missing # mental health conditions	.048 (− .025; .12)	.196	.009 (− .088; .105)	.859	.075 (− .038; .187)	.193	− .152 (− .34; .036)	.113	.017 (− .069; .102)	.703
Year of the survey										
2018 reference										
2019	.033 (− .016; .082)	.183	.014 (− .038; .066)	.591	.011 (− .059; .081)	.754	− .009 (− .076; .059)	.804	− .008 (− .06; .044)	.763
2020	.01 (− .048; .068)	.736	− .003 (− .065; .06)	.936	− .047 (− .135; .04)	.29	− .05 (− .131; .03)	.219	− .021 (− .08; .038)	.487
2021	− .011 (− .059; .036)	.633	− .028 (− .078; .022)	.277	− .046 (− .11; .019)	.165	− .011 (− .069; .048)	.721	.018 (− .02; .057)	.353

Note: all models are estimated as multivariate logistic regressions

^a^AME of going from 25 to 75th percentile of Gini

^b^AME of going from 25 to 75th percentile of census tract poverty rate

## Discussion

Consistent with our first hypothesis, the findings show that both lower household income (below $50,000) and middle household income ($50,000–$89,000) cancer survivors had a higher risk of financial hardship compared to high household income survivors. In the context of the state of New Jersey, this means that cancer survivors residing in households in the lower two income quartiles have a higher risk of financial hardship compared to cancer survivors in the top two household income quartiles. The findings on middle-income cancer survivors are novel and suggest that beyond the effect of cancer care costs on cancer care outcomes, financial hardship associated with treatment can adversely impact dental, eye, and mental health care utilization among both cancer survivors with low and middle household income. The higher rates of forgoing dental and vision care may also reflect a lack of insurance coverage for these services, even among those with private insurance and Medicare, which can result in high out-of-pocket costs. Most existing research on financial hardship among cancer survivors [27] focuses on the role that low household income and poverty play in financial hardship, and our findings expand our understanding of the role household income and county income inequality play in shaping financial hardship.

We found mixed results for our second hypothesis. The relationship between income inequality at the county level and financial hardship was significant for some measures of material conditions and coping behaviors, but not all. Specifically, higher income inequality was linked to an increased risk in one out of three material conditions and in two out of five coping behavior measures. Additionally, greater income inequality was associated with an increased risk of worry about large medical bills that reflected the psychological response dimension of financial hardship. To the best of our knowledge, this is the first study of cancer survivors that examines the association of financial hardship and area-level income inequality.

Taken together our findings indicate that the risk of financial hardship among cancer survivors could be shaped not only by their household income but also by the distribution of income within their communities. Cancer survivors residing in counties with greater redistribution of total county income away from poorer to richer households may be at a higher risk of financial hardship. However, given the mixed nature of our findings, more research is needed to better understand the role area-level income inequality may play in shaping the financial hardship of cancer survivors.

### Study implications

Our results have implications for future research and clinical practice. In terms of research implications, evaluating middle household income cancer survivors as a separate population from high household income survivors as well as assessing income inequality as a factor in financial hardship may provide important information. Future research should further examine the role income inequality may play in shaping the financial hardship of cancer survivors and explore mechanisms behind this relationship, including the availability and utilization of social programs, community resources, and social differentiation [19, 21]. Such studies can inform communities, particularly communities with levels of income inequality how to best address the needs of cancer survivors in these communities. In terms of clinical implications, it will be important to include middle household income survivors in financial navigation and mitigation programs. In addition, our results underscore that financial hardship among cancer survivors can have far-reaching consequences inflicting delays in a broad range of important health care services outside of cancer care, including dental care, eye care, and mental health care. These issues are often overlooked; even by cancer centers that offer comprehensive financial hardship screening and navigation, assistance with paying for health care services outside of cancer care is typically not available [28].

### Strengths and limitations

To our knowledge, this is the first study that examines the relationship between income inequality and financial hardship among cancer survivors. We evaluated multiple dimensions of financial hardship, including coping behaviors of delaying or forgoing both cancer-related and non-cancer medical care that are often overlooked in the existing literature. Our focus on an entire state is a strength because most of the existing literature uses either national data or state-level data that is not statewide (i.e., from only a part of the state).

In terms of limitations, the proportion of observations with missing data on household income was relatively high, which may bias the estimation results, although the presence of census tract poverty rate in the data analysis is likely to be partially accounted for missing data. We were unable to account for the effects of household assets, including liquid assets, which is a common problem in financial hardship literature [8, 11]. Our sample only included survivors who were able to read and speak English, which limits generalizability in future research. Finally, the cross-sectional design implies that estimation results indicate associations and may not necessarily imply causal relationships.

## Conclusions

Both low household income and middle household income cancer survivors are at increased risk of certain aspects of financial hardship, as are cancer survivors residing in communities with higher income inequality. Failing to assess and address rising income inequality and stagnant incomes of low-income and middle-income households may exacerbate financial hardship experienced by cancer survivors and compromise not only their cancer care utilization, but also non-cancer health care utilization.

## Data Availability

The data that support the findings of this study are available on request from the corresponding author. The data are not publicly available due to privacy or ethical restrictions.
